# Social Media and Rating Sites as Tools to Understanding Quality of Care: A Scoping Review

**DOI:** 10.2196/jmir.3024

**Published:** 2014-02-20

**Authors:** Lise M Verhoef, Tom H Van de Belt, Lucien JLPG Engelen, Lisette Schoonhoven, Rudolf B Kool

**Affiliations:** ^1^IQ healthcareRadboud University Medical CenterNijmegenNetherlands; ^2^Radboud REshape & Innovation CenterRadboud University Medical CenterNijmegenNetherlands; ^3^Emergency Healthcare NetworkRadboud University Medical CenterNijmegenNetherlands; ^4^Faculty of Health SciencesUniversity of SouthamptonSouthamptonUnited Kingdom

**Keywords:** social media, rating sites, patient experiences, patient satisfaction, quality of health care

## Abstract

**Background:**

Insight into the quality of health care is important for any stakeholder including patients, professionals, and governments. In light of a patient-centered approach, it is essential to assess the quality of health care from a patient’s perspective, which is commonly done with surveys or focus groups. Unfortunately, these “traditional” methods have significant limitations that include social desirability bias, a time lag between experience and measurement, and difficulty reaching large groups of people. Information on social media could be of value to overcoming these limitations, since these new media are easy to use and are used by the majority of the population. Furthermore, an increasing number of people share health care experiences online or rate the quality of their health care provider on physician rating sites. The question is whether this information is relevant to determining or predicting the quality of health care.

**Objective:**

The goal of our research was to systematically analyze the relation between information shared on social media and quality of care.

**Methods:**

We performed a scoping review with the following goals: (1) to map the literature on the association between social media and quality of care, (2) to identify different mechanisms of this relationship, and (3) to determine a more detailed agenda for this relatively new research area. A recognized scoping review methodology was used. We developed a search strategy based on four themes: social media, patient experience, quality, and health care. Four online scientific databases were searched, articles were screened, and data extracted. Results related to the research question were described and categorized according to type of social media. Furthermore, national and international stakeholders were consulted throughout the study, to discuss and interpret results.

**Results:**

Twenty-nine articles were included, of which 21 were concerned with health care rating sites. Several studies indicate a relationship between information on social media and quality of health care. However, some drawbacks exist, especially regarding the use of rating sites. For example, since rating is anonymous, rating values are not risk adjusted and therefore vulnerable to fraud. Also, ratings are often based on only a few reviews and are predominantly positive. Furthermore, people providing feedback on health care via social media are presumably not always representative for the patient population.

**Conclusions:**

Social media and particularly rating sites are an interesting new source of information about quality of care from the patient’s perspective. This new source should be used to complement traditional methods, since measuring quality of care via social media has other, but not less serious, limitations. Future research should explore whether social media are suitable in practice for patients, health insurers, and governments to help them judge the quality performance of professionals and organizations.

## Introduction

Several studies have shown significant variation in the quality of care delivered by health care providers and individual professionals [1,2]. Insight into quality of care—and especially information about the differences between providers—is important as it allows stakeholders, including consumers, health insurers, and governmental organizations such as health care inspectorates, to compare care providers and choose between them [3].

Patient centeredness is an important part of quality in health care that has gained more attention since the Institute of Medicine published its report on improving health care quality in 2001 [4]. Patient values, needs, and preferences should be respected and should guide clinical decisions. Therefore, it is essential to gain insight into quality of care from a patient’s perspective. This can be achieved using traditional methods such as surveys, panels, or focus groups. Notwithstanding the potential of these strategies, they also have serious limitations [5]. First, there are several methodological challenges such as social desirability bias and selection bias [6-8]. This means that patients might give answers they think are socially accepted rather than being strictly honest (social desirability bias) or that patients who are questioned are not representative of the whole patient population (selection bias). Second, there is a time lag between the experience and the information given to the organization, insurer, patients, or health care inspectorate. Since focus groups and surveys do not allow patients to share their feedback directly after the experience, bias may occur. Third, it is difficult to reach large groups of people [9,10], and some specific groups such as ethnic minorities and people with low literacy are often not included.

Information on social media could be of value to overcome these limitations, since these new media are easy to use and are used by the majority of the population. However, people using social media are not necessarily representative of the whole population, since, for example, elderly and ethnic minorities are underrepresented in Internet use [11]. Social media are a group of Internet-based applications that build on the ideological and technological foundations of Web 2.0 and that allow the creation and exchange of user-generated content [12]. The popularity of social media can be explained by four major characteristics: they connect, create, consume, and control (online reputation) [13]. A huge and still increasing number of people use social media. For example, more than 1 billion people worldwide use Facebook, 200 million people use Twitter [14], and the number of ratings on health care rating sites has increased consistently in the past few years [15,16].

Rating sites are not a new phenomenon in our society. Many people have been using these sites to rate and find services for several years. Examples are Yelp (restaurants) and TripAdvisor (travel). In health care, rating sites allow people to share their opinion about health care providers or professionals. They are a modern way to identify what patients think and feel about health care [17]. This collection of patient experiences within health care on the Internet has been described as “crowd validation of patient experience” by Cambria et al and as a “cloud of patient experience” by Greaves et al [18,19]. Since ratings of large and complex health care services such as hospitals are hard to interpret, websites that rate individual doctors, physician-rating sites (PRSs), are a promising type of rating site. Despite resistance from the medical profession, PRSs are growing consistently [16,19].

Since many people use social media to share their experiences with health care, social media could help create transparency in the quality of health care from the patient’s perspective. For example, Timian et al investigated the number of “likes” on the Facebook pages of 40 American hospitals [20]. They found that this number was negatively associated with 30-day mortality and positively with patient recommendations, which indicates a correlation between information on social media and quality of care. This example shows that social media can provide useful information about quality of care. Social networks such as Facebook and Google+, might provide information like comments, “likes”, or “+1”s on the page of a hospital. Patients’ experiences in health care might be shared on discussion forums or patient networks. Even microblogs, like Twitter, could function as a source of information about quality of care, although these short, unstructured messages contain minimal information [19].

The number of studies showing the information value of social media for quality of health care is growing rapidly. This has created a need for a systematic synthesis concerning the relation between social media and quality of care, its usefulness, and potential effects. Therefore, we performed a scoping review with the following goals: (1) to map the literature on the association between social media and quality of care, (2) to identify different mechanisms of this relationship, and (3) to determine a more detailed agenda for this relatively new research area.

## Methods

### Framework

For this study, we used the framework of Arksey and O’Malley for scoping reviews, further developed by Levac et al and Daudt et al [21-23]. A scoping study is a method to quickly map the evidence of a particular field [21]. More specifically, Mays et al defined it as follows: “to map rapidly the key concepts underpinning a research area and the main sources and types of evidence available, and can be undertaken as standalone projects in their own right, especially where an area is complex or has not been reviewed comprehensively before” [22]. It is therefore the preferred method in this study since it concerns a relatively broad issue that has not yet been clearly defined in the literature. We followed the six steps of the framework: (1) identifying the research question, (2) identifying relevant studies, (3) selecting studies, (4) charting the data, (5) collating, summarizing, and reporting the results, and (6) stakeholder consultation. The sixth step was followed throughout the study, as suggested by Daudt et al [23]. Since the research area studied in this review concerned only observational studies and is relatively new, a formal quality assessment of the included studies was not performed. However, we identified the different study designs and reported them as part of the results. The six steps of the framework will be discussed below.

### Step 1: Identifying the Research Question

This scoping review focused on the association between information from patients on social media and quality of care. The research question was “What is the association between information from patients, clients, and their relatives on various types of social media and the quality of health care?” We defined information from patients, clients, and relatives on social media as any information about health care providers, health care professionals, or about the health care system in general, shared via online social media such as rating sites (rating of health care providers or professionals), (micro)blogs, social network sites, and forums. The working definition formulated by the Institute of Medicine was used to define quality of care [4]. They propose a broad definition in which good quality health care is determined by six aims: health care should be safe, effective, patient-centered, timely, efficient, and equitable.

### Step 2: Identifying Relevant Studies

To identify relevant studies, we used a two-step search strategy. First, we conducted a preliminary search in PubMed to identify key articles. This step was important since this research topic is new, and little was known about relevant keywords and MeSH (Medical Subject Headings) terms. The search strategy was developed by 2 authors with expertise in performing systematic reviews (LV and RK) and further improved by an author with expertise in social media for health care (TB). The search resulted in 17 key articles.

The second step consisted of reshaping the search strategy. It was peer-reviewed by an information specialist employed at the medical library of our university hospital. A standardized list of criteria for assessing searches in the academic literature was used [24]. The final search strategy was built on four themes: social media, patient experience, quality, and health care. For every theme, thesaurus terms and text words in title and abstract were used. The themes were combined as follows: “social media” AND (“patient experience” OR “quality”) AND “health care”. Multimedia Appendix 1 shows the search strategies for the final search.

We searched four electronic databases for relevant articles: PubMed, Embase, CINAHL, and Web of Science. Since we aimed to give a broad overview of existing literature, we did not restrict the number of articles by setting limits for date of publication, type of article, or language. Additionally, we screened reference lists of included articles for relevant studies and invited several experts working in this field to share relevant articles.

### Step 3: Selecting Studies

Articles were independently reviewed and scored by 2 authors (RK, LV) using title and abstract. Disagreements were discussed until consensus was reached. Finally, full texts were reviewed to determine if the articles were eligible for inclusion in the review.

For inclusion, articles should concern information from patients, clients, or their relatives on social media and the relation to quality of health care. Articles were excluded when no abstract or full text was available. A few examples of excluded articles were articles about quality improvement using a social media application (not about relation to quality of health care), articles addressing Web-based surveys about quality of health care (not about social media), and articles concerning the use of social media by medical professionals (not about information from patients, clients, or their relatives).

### Step 4: Charting the Data

A data extraction form was developed by the different authors together, to ensure the approach was consistent with the research question and purpose of the scoping review. Key elements that were extracted from the articles were journal, type of study, country, type of social media application, objective(s), conclusions, and a subjective assessment of the attitude of the authors towards the relation between social media and quality of health care (“positive”, “positive with reservations”, or “negative”). To ensure that all relevant data were extracted according to the research question, all articles were assessed, and data were extracted independently by 2 researchers (LV, TB).

### Step 5: Collating, Summarizing, and Reporting the Results

As proposed by Levac et al [21], we identified three distinct steps in this phase. First, we analyzed the data from the included articles and reported general characteristics. Second, the results related to the research question were described. Thus, information about the association between information on social media and quality of health care was summarized. These results were categorized by type of social media application described in the different articles. Third, the results were discussed and implications for further research, practice, and policy were described.

### Step 6: Stakeholder Consultation

Professionals from the Health Care Inspectorate (the Netherlands), the Care Quality Commission (England), and several Dutch inspectorates outside of the health care sector were consulted during the process. Examples of inspectorates in other sectors were the Dutch Tax Administration (Belastingdienst) and the Dutch Inspectorate of Education (Onderwijsinspectie). Preliminary results from this review were shared, and suggestions and advice were used to improve this scoping review.

## Results

### General Information

**Figure 1:**
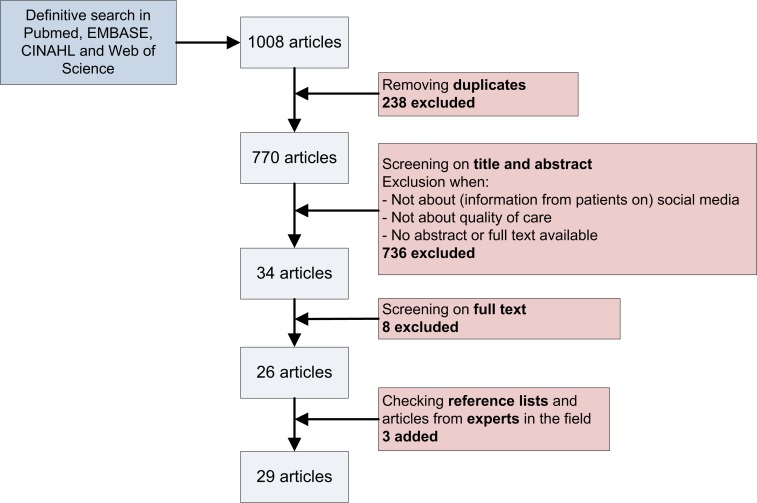
Study selection process.

Our preliminary PubMed search resulted in 610 hits. Of these, 17 articles were labeled as key articles [3,15,19,20,25-37]. Our final search in PubMed, EMBASE, CINAHL, and Web of Science resulted in 392, 488, 55, and 73 articles respectively, totaling 1008 studies. After removing duplicates, 770 studies remained. After screening on title and abstract and reading full texts, 26 articles were included for this review. Another 3 articles were added after screening reference lists of included articles and inviting experts in the field to share relevant articles [3,15,19,20,25-49]. Figure 1 gives an overview of the study selection process.

A description of the included papers is provided in Table 1. The studies included in this review were mainly performed in the United States (n=10) or the United Kingdom (n=7). The other studies were conducted in Germany, the Netherlands, Taiwan, and Peru. Some studies did not explicitly state in which country/countries they were performed. Most articles focused on health care rating sites (n=21). Three studies concentrated on Facebook in particular, where other studies addressed social media in general. Of the 29 articles, 15 described original research. The others articles were six opinion papers, two reviews, two editorials, a news item, an essay, and two pieces of correspondence. Regarding the attitude of the authors towards the relation between social media and quality of health care, 7 authors were positive, 20 were positive but with reservations, and 2 were negative. In general, the articles could be divided according to social media application, which resulted in three groups: rating sites, Facebook, and social media in general.

**Table 1:** Description of the included studies.

No.	Reference	Journal	Type of study	Country	Type of social media^a^	Attitude^b^
1	Abdul, 2011 [38]	Lancet	Correspondence	Taiwan	Facebook	+
2	Adams, 2010 [25]	Int J Med Inf	Review	Netherlands	Social media in general	+/–
3	Adams, 2011 [26]	Soc Sci Med	Original	United States, United Kingdom, Netherlands	Rating sites	+/–
4	Bacon, 2009 [39]	BMJ	Opinion paper	United Kingdom	Rating sites	+
5	Bardach, 2012 [40]	BMJ Qual Saf	Original	United States	Rating sites	+
6	Black, 2009 [41]	Inform Prim Care	Original	United States	Rating sites	+/–
7	Denecke, 2013 [42]	Methods Inf Med	Editorial	n/a	Social media in general	+/–
8	Emmert, 2012 [27]	Methods Inf Med	Original	Germany	Rating sites	+/–
9	Emmert, 2013 [43]	J Med Internet Res	Review	n/a	Rating sites	+/–
10	Galizzi, 2012 [44]	BMJ Open	Original	United Kingdom	Rating sites	+/–
11	Gao, 2012 [15]	J Med Internet Res	Original	United States	Rating sites	+/–
12	Greaves, 2012^c^[28]	BMJ Qual Saf	Original	United Kingdom	Rating sites	+
13	Greaves, 2012 [29]	Arch Intern Med	Original	United Kingdom	Rating sites	+/–
14	Greaves, 2012 [30]	J Med Internet Res	Original	United Kingdom	Rating sites	+/–
15	Greaves, 2013 [19]	BMJ Qual Saf	Opinion paper	United Kingdom	Social media in general	+/–
16	Hammond, 2008 [45]	Guidelines in Practice	Opinion paper	n/a	Rating sites	+
17	Kadry, 2011 [31]	J Med Internet Res	Original	United States	Rating sites	+/–
18	Lagu, 2010 [32]	J Gen Intern Med	Original	United States	Rating sites	+/–
19	Lopez, 2012 [46]	J Gen Intern Med	Original	United States	Rating sites	+/–
20	McCartney, 2009 [47]	BMJ	Opinion paper	United Kingdom	Rating sites	–
21	Reimann, 2010 [33]	BMC Health Serv Res	Original	n/a	Rating sites	+/–
22	Rozenblum, 2013 [48]	BMJ Qual Saf	Editorial	n/a	Social media in general	+
23	Segal, 2012 [34]	J Med Internet Res	Original	United States	Rating sites	+/–
24	Strech, 2011 [35]	J Med Internet Res	Opinion paper	n/a	Rating sites	+/–
25	Tanne, 2013 [36]	BMJ	News item	United States	Rating sites	+/–
26	Tello, 2013 [49]	Am J Med Qual	Correspondence	Peru	Facebook	–
27	Thielst, 2011 [37]	Front Health Serv Manage	Opinion paper	United States	Social media in general	+
28	Timian, 2013 [20]	Am J Med Qual	Original	United States	Facebook	+
29	Trigg, 2011 [3]	J Health Serv Res Policy	Essay	n/a	Rating sites	+/–

^a^Rating sites: various types of health care rating sites like physician rating sites or hospital rating sites.

^b^A subjective assessment of the authors’ attitude towards the relation between social media and quality of health care (+: positive, +/–: positive with reservations, –: negative).

^c^We refer to three different papers by Greaves et al in 2012: see citations [28-30].

### Association Between Types of Social Media and Quality of Care

#### Rating Sites

##### Association Between Ratings and Quality of Care

Most identified studies (21/29) concerned the association between ratings on rating sites and quality of care. Table 2 shows the correlations that have or have not been shown in various original studies. Greaves et al demonstrated a correlation between Web-based patient ratings of hospitals (on NHS Choices) and conventional surveys of patient experiences. Furthermore they showed a relationship between these ratings and objective measures of quality, including readmission rates, mortality, and infection rates. They investigated the same associations with ratings of family physician practices on NHS choices. These ratings are moderately associated with measures of patient experience and weakly with clinical quality [28-30]. A study into the rating of physicians on the RateMDs website suggests that these ratings correlate positively with physician quality, measured by board certification, education, and malpractice claims. The ratings were based on a small number of reviews, and most rating variation reflected evaluations of punctuality and staff. The authors concluded that further research is needed into the correlation between ratings and clinical outcomes [15]. Segal et al found that while the total number of reviews correlated with surgeon volume (as a proxy for surgeon quality), the actual rating value did not [34]. Bardach et al describe a correlation between hospital scores on Yelp (a commercial rating website) and a more traditional measure of patient experience, the Hospital Consumer Assessment of Healthcare Providers and Systems (HCAHPS) scores. Furthermore, they found correlations between Yelp scores and mortality and readmission rates. According to the authors, their data suggests that consumers posting ratings on Yelp may observe aspects of care related to important patient outcomes [40].

Next to these original studies, three other studies argue that information from rating sites reflects quality of care. Hammond (2008), broadcaster and general practitioner, thinks the safety is in numbers: “If you can’t find 200 patients who approve of what you’re doing, you’re in the wrong job!” [45]. Bacon, physician and shareholder of the rating site, iWantGreatCare, argues that rating sites provide valid, detailed, and timely feedback that is needed to efficiently measure quality and satisfaction. Therefore, professionally responsible rating sites will increase standards of care but only for those organizations and doctors that think the experience of the patient is as important as excellent clinical outcome [39]. Finally, Trigg, researcher in the field of health care and social care, focuses on the use of PRSs by patients. She states that the increasing use of these sites suggests that patients seek new ways to give feedback on care providers. Understanding the reasons for use of PRSs can give insight into how the information can be used for quality improvement [3].

In contrast with the studies mentioned above, various studies suggest that patient ratings are not, or not yet, useful enough to give insight into the quality of care for the following reasons.

**Table 2:** Correlations between information from social media and measures of quality^a^.

		Measure of quality								
Article	Info from social media	Patient experiences	Readmission rates (different measures used)	Mortality (different measures used)	Board certification	Education	Malpractice claims	Infection rates	Clinical quality indicators	Surgeon volume
Bardach, 2012 [40]	Hospital rating	+	+	+/–						
Gao, 2012 [15]	Physician rating				+	+	+/–			
Greaves, 2012 [28,29]	Hospital rating	+	+	+/–				+		
Greaves, 2012 [30]	Family physician rating	+							+/–	
Segal, 2012 [34]	No. of reviews									+
	Rating value									–
Timian, 2013 [20]	No. of “likes”	+		+						

^a^This table presents the correlations/associations as stated by the authors in the various papers (+: there is correlation, +/–: correlation is weak or not found for all aspects, –: there is no correlation).

##### Partial Quality Measurement

Reimann et al performed a study to investigate the extent to which English- and German-language PRSs represented different dimensions of patient experience and satisfaction, determined by a systematic review. They identified 13 dimensions in three categories: characteristics of encounter between doctor and patient (eg, trust), organizational aspects (eg, accessibility), and overarching assessment categories (eg, general satisfaction). They found that none of the 21 investigated PRSs represented all 13 dimensions. However, the three most visited German sites represented between 8 and 11 dimensions. The three most trafficked English-language PRSs represented between 5 and 6 dimensions. Specifically the dimensions communication skills and information/advice were missing [33].

##### Influence of Patient Characteristics on Ratings

Emmert et al state that patient satisfaction and outcome measures on PRSs are not risk-adjusted [27], although research has shown that patient satisfaction results are influenced by age, education, and health status [6]. Also, Galizzi et al found that subjects who give feedback on doctor-ranking websites are unlikely to be representative of the overall patient pool. This indicates that it is important to look at user characteristics when interpreting results from doctor-rating sites [44].

##### Positive Sentiment of Ratings

Four original studies analyzed the content of reviews on rating sites. Black et al analyzed 16,703 reviews on 6101 providers in the United States. They found that online ratings were largely positive [41]. This was also found by Lagu et al for physician ratings. The study also identified narratives that appeared to be written by physicians themselves [32]. Kadry et al performed an analysis of 4999 online physician ratings. They concluded that most patients give physicians a favorable rating [31]. Finally, Lopez et al analyzed 712 reviews of primary care physicians. The majority of these reviews was positive [46].

##### Factors Other Than Quality of Care on Ratings

Adams et al performed an analysis of four share-your-experience websites in three countries, supplemented by interviews with stakeholders from the Netherlands (website developers, hospitals, insurers, and members of the Dutch Health Inspectorate). Their results show that the sharing of experiences by patients is not automatic but encouraged by website creators who have their own purpose with these posts [26]. Lopez et al conclude that “patient reviews are affected by more aspects of care than the patient-physician interaction only. Accessibility, convenience and staff also play a role” [46].

##### Low Number of Reviews

A study looking at the ratings of 500 randomly selected urologists on 10 rating websites showed an average of 2.4 ratings per doctor. According to Tanne et al, this indicates that these sites need more reviews to make them more reliable [36].

##### Ratings and the Potential Harm to a Physician’s Reputation

Strech et al addressed the ethical discussion around the basic concept of PRSs. They conclude that the potential harms for physicians that can result from PRSs (financial and psychological) need to be contained without limiting the potential benefits for patients with respect to health, health literacy, and equity [35]. McCartney, a general practitioner, goes one step further. She argues against the use of rating sites in health care because “it is a non-evidence-based intervention with potentially damaging strings attached”. For example, some medical work, like child-protection and psychiatry, has the constant potential for conflict. Also, factors like socioeconomic status might influence satisfaction with general practice services [47]. Emmert et al performed a systematic review of the literature about physician rating sites. They conclude that rating sites are gaining more attention in research and mention several shortcomings of these sites from literature. Examples include the fact that it is often not possible to relate anonymous feedback to specific incidents, making it unlikely that care providers can learn from the comments. Also, anonymous ratings makes it easy to abuse these sites, which might lead to defamation of professionals or misinformation to patients [43].

#### Facebook

Three articles focused in particular on the social network site Facebook. A study by Timian et al, involving 40 hospitals in the United States, found that the number of “Likes” on the Facebook page of the hospitals had a negative association with the 30-day mortality rate and a positive association with patient recommendation [20]. Tello et al commented on this article by stating that measuring quality of care with Facebook likes in Peru is confronted with several barriers. For example, people on Facebook are hardly representative of the patient population since only a small proportion of Peruvians, and mostly younger people, use Facebook [49]. Next to this, Abdul describes how Facebook enabled collaboration between stakeholders in emergency-medicine policy in Taiwan. An active discussion on a Facebook group about overcrowded emergency rooms was followed by the Minister of Health’s involvement, which eventually led to health care reforms in the country [38].

#### Social Media in General

Five articles focused on social media in general, without handling one type in particular. Adams et al performed a literature and Web review on the reliability of online health information in light of the increasingly popular Web 2.0. They state that issues about reliability, like disclosure of authorship and privacy, should not easily be dismissed. Therefore, caution is required when newly popular Web applications are used for health purposes [25]. Thielst discusses the use of social media in health care. She argues that social media platforms are a cheap way for health care organizations to hear the voice of patients and get feedback on their care [37]. Furthermore, an editorial by Denecke et al reports that information from medical social media could provide a new source of information. For example, patient stories on discussion forums could enable earlier detection of adverse drug effects [42]. Rozenblum et al also emphasize the growing importance of patients’ experience acquired from social media. They think that this information will complement traditional patient surveys and will help identify poor care and outstanding care [48]. Finally, Greaves et al describe the possibility of using the “cloud of patient experience” on the Internet for detection of poor quality care. They provide advantages and disadvantages of different sources of information (eg, rating sites, patient forums, social networks) and name several technical and logistic limitations for using and processing information about quality of care from social media. The authors suggest the comparison between conventional measures of patient experience with information from the online cloud of patient experiences (after collection and processing) in future research [19].

## Discussion

### Principal Findings

This review showed that, although literature about the topic is limited, several studies indicate a relation between information on social media and quality of health care. Interestingly, most of these studies concern rating sites. An association was found for ratings of whole organizations as well as for individual physicians, although different measures for quality of care were used. These findings show that social media, and especially rating sites, could be a fast and efficient way to gather information about quality of care. However, several disadvantages of using social media also exist. In this discussion, we will put our findings in perspective.

This scoping review identified studies in which subjective ratings were not only correlated to subjective measures of quality but also to objective measures. Patient rating of a certain hospital is likely to correlate with patient recommendation. Since patients are not likely to have insight into hospital mortality or infection rates, the associations shown by for example Timian et al [20] and Greaves et al [29] are remarkable. The fact that these patient ratings correlate to (aspects of) quality of health care might make expensive, traditional measures of patient experiences unnecessary in the future. However, associations in the included studies were shown only at one point in time. Research is also needed into the predictive value of ratings over time [19]. This feature is especially of interest for supervisory bodies such as health care inspectorates. Therefore, it is important to perform studies with a longitudinal design. When looking at different purposes for gathering information about quality of health care, social media might be useful as a predictor for low quality health care, which has already been shown by Google flu trends. This sophisticated tool from Google analyzes health care-related search queries from people worldwide in their search engine. Because there is a close relationship between the number of people searching for influenza-related topics and those who have influenza symptoms, this tool can predict flu outbreaks much faster than conventional surveillance [50]. Furthermore, new techniques are being developed to analyze unstructured data about the quality of health care on the Internet. Greaves et al showed that sentiment analysis of patients’ comments about their health care is possible and reasonably accurate [51].

In contrast with these findings, the articles included in this study also identified several drawbacks concerning rating sites. These include the fact that rating is often anonymous and as a result, rating values are not risk-adjusted and are vulnerable to fraud. A health care professional can, for example, rate him/herself or colleagues. Also, ratings are often based on only a few reviews and are predominantly positive. Furthermore, people providing feedback on health care via social media are presumably not always representative of the patient population. Further, reviews from patients are influenced by other factors than quality of care. Not only the patient-physician relation is rated but issues like accessibility play a role too. Therefore, several authors suggest that information from social media should be used with caution. Also, several examples exist in the Netherlands where information on new media is biased. A popular Dutch opinion website has influenced several polls by encouraging people to vote for a certain answer [52]. We are also aware of a case where media attention around a poorly performing hospital elicited more positive reviews shared by people who wanted to stand up for this hospital. This shows that groups of people can purposely influence information on social media. Another important issue is that there are many organizational differences between rating sites that can influence the content presented. Examples are the presence or absence of editors that check ratings before they appear on the site and the possibility for doctors to share their views on comments [53]. Also, it should be realized that rating sites can be owned by stakeholders that may have conflicts or interests. Sometimes these sites are organized by the government, such as NHS Choices, and sometimes these sites can be privately owned or owned by patient federations such as ZorgkaartNederland in the Netherlands.

### Limitations

Our study has some limitations. It is possible that publication bias has affected our results since studies without significant results are often not published. However, we found many articles that also discussed the negative aspects of using social media to gain insight into quality of health care. This suggests that influence of publication bias was minimal. Furthermore, our study was restricted to literature from online databases. Future studies might consider inclusion of grey literature.

### Conclusion

Social media and rating sites in particular are an interesting new source of information about quality of care. However, this new source should, at least for now, be used to complement traditional methods, since measuring quality of care via social media has other, but not less serious, limitations [28]. Future research should focus on comparing objective traditional measures of quality with subjective information from social media, which has also been suggested by other authors [17,54]. This will provide more evidence on the association between the two approaches. Furthermore, this scoping review provides a basis for a more systematic review of the literature, which can give a more definite answer about how information from social media can be used to assess quality of health care.

## Multimedia Appendix 1

Search strategies for the different online databases.

## Abbreviations

HCAHPS: Hospital Consumer Assessment of Healthcare Providers and Systems
MeSH: medical subject headings
NHS: National Health Service
NWO: Netherlands Organisation for Scientific Research
PRS: physician-rating sites
ZonMw: The Netherlands Organisation for Health Research and Development
