# Supplementary material for: Social Media and Rating Sites as Tools to Understanding Quality of Care: A Scoping Review
Source: J Med Internet Res. 2014 Feb 20;16(2):e56. doi: 10.2196/jmir.3024 (PMC3961699; doi:10.2196/jmir.3024)
Supplement: Supplementary file 1 [file jmir_v16i2e56_app1.pdf]

Database	Search strategy (searched at 04-07-2013)
**PubMed**<br>392 hits	("Social Media"[Mesh] OR "Blogging"[Mesh] OR social media[tiab] OR blog*[tiab] OR microblog*[tiab] OR wiki*[tiab] OR flickr[tiab] OR youtube[tiab] OR twitter[tiab] OR tweet*[tiab] OR facebook[tiab] OR myspace[tiab] OR "web 2.0"[tiab] OR "web 3.0"[tiab] OR "medicine 2.0"[tiab] OR "health 2.0"[tiab] OR crowdsourcing[tiab] OR online rating*[tiab] OR rating website*[tiab] OR rating site*[tiab] OR hospital rating*[tiab] OR patient opinion website*[tiab]) AND (("Quality of Health Care"[Mesh] OR quality[tiab]) OR ("Consumer Satisfaction"[Mesh] OR patients rating*[tiab] OR patient rating*[tiab] OR patients' rating*[tiab] OR patient's rating*[tiab] OR patient satisfaction[tiab] OR patients satisfaction[tiab] OR patient's satisfaction[tiab] OR patient experience*[tiab] OR patients experience*[tiab] OR patients' experience*[tiab] OR patient's experience*[tiab] OR patient opinion[tiab] OR patients opinion[tiab] OR patient's opinion[tiab] OR consumer satisfaction[tiab] OR consumer preference*[tiab])) AND ("Delivery of Health Care"[Mesh] OR healthcare[tiab] OR health care[tiab])
**Web of Science**<br>73 hits	("social media" OR blog* OR microblog* OR wiki* OR flickr OR youtube OR twitter OR tweet* OR facebook OR myspace OR "web 2.0" OR "web 3.0" OR "medicine 2.0" OR "health 2.0" OR crowdsourcing OR "online rating*" OR "rating website*" OR "rating site*" OR "hospital rating*" OR "patient opinion website*") AND (quality OR ("patients rating*" OR "patient rating*" OR "patients' rating*" OR "patient's rating*" OR "patient satisfaction" OR "patients satisfaction" OR "patient's satisfaction" OR "patient experience*" OR "patients experience*" OR "patients' experience*" OR "patient's experience*" OR "patient opinion" OR "patients opinion" OR "patient's opinion" OR "consumer satisfaction" OR "consumer preference*")) AND (healthcare OR "health care")
**EMBASE 1974 to 2013 July 3**<br>488 hits	(social media/ OR "social media".ti,ab. OR blog*.ti,ab. OR microblog*.ti,ab. OR wiki*.ti,ab. OR flickr.ti,ab. OR youtube.ti,ab. OR twitter.ti,ab. OR tweet*.ti,ab. OR facebook.ti,ab. OR myspace.ti,ab. OR "web 2.0".ti,ab. OR "web 3.0".ti,ab. OR "medicine 2.0".ti,ab. OR "health 2.0".ti,ab. OR crowdsourcing.ti,ab. OR "online rating*".ti,ab. OR "rating website*".ti,ab. OR "rating site*".ti,ab. OR "hospital rating*".ti,ab. OR "patient opinion website*".ti,ab.) AND ((exp health care quality/ OR quality.ti,ab.) OR (exp patient attitude/ OR "patient rating*".ti,ab. OR "patients rating*".ti,ab. OR "patients' rating*".ti,ab. OR "patient's rating*".ti,ab. OR "patient satisfaction".ti,ab. OR "patients satisfaction".ti,ab. OR "patient's satisfaction".ti,ab OR "patient experience*".ti,ab. OR "patients experience*".ti,ab. OR "patients' experience*".ti,ab. OR "patient's experience*".ti,ab. OR "patient opinion".ti,ab. OR "patients opinion".ti,ab. OR "patient's opinion".ti,ab. OR "consumer satisfaction".ti,ab. OR "consumer preference*".ti,ab.)) AND (exp health care/ OR healthcare.ti,ab. OR "health care".ti,ab.)
**CINAHL**<br>55 hits	((MH "Social Media") OR TI ("social media" OR blog* OR microblog* OR wiki* OR flickr OR youtube OR twitter OR tweet* OR facebook OR myspace OR "web 2.0" OR "web 3.0" OR "medicine 2.0" OR "health 2.0" OR crowdsourcing OR "online rating*" OR "rating website*" OR "rating site*" OR "hospital rating*" OR "patient opinion website*") OR AB ("social media" OR blog* OR microblog* OR wiki* OR flickr OR youtube OR twitter OR tweet* OR facebook OR myspace OR "web 2.0" OR "web 3.0" OR "medicine 2.0" OR "health 2.0" OR crowdsourcing OR "online rating*" OR "rating website*" OR "rating site*" OR "hospital rating*" OR "patient opinion website*")) AND (((MH "Quality of Health Care+") OR TI quality OR AB quality) OR ((MH "Patient Satisfaction") OR TI ("patient rating*" OR "patients rating*" OR "patient's rating*" OR "patients' rating*" OR "patient satisfaction" OR "patients satisfaction" OR "patient's satisfaction" OR "patient experience" OR "patients experience*" OR "patient's experience*" OR "patients' experience*" OR "patient opinion" OR "patients opinion" OR "patient's opinion" OR "consumer satisfaction" OR "consumer preference*") OR AB ("patient rating*" OR "patients rating*" OR "patient's rating*" OR "patients' rating*" OR "patient satisfaction" OR "patients satisfaction" OR "patient's satisfaction" OR "patient experience" OR "patients experience*" OR "patient's experience*" OR "patients' experience*" OR "patient opinion" OR "patients opinion" OR "patient's opinion" OR "consumer satisfaction" OR "consumer preference*"))) AND ((MH "Health Care Delivery+") OR TI (healthcare OR "health care") OR AB (healthcare OR "health care"))
